# Comparison of the levels of depression and anxiety in elderly aortic stenosis patients treated with surgical or transcatheter aortic valve replacement

**DOI:** 10.1186/s13019-022-01888-6

**Published:** 2022-06-03

**Authors:** Jiao Sun, Qing-Tao Meng, Yu-Wei Wang, Wei-Long Zhao, Feng-Zhi Sun, Ji-Hong Liu, Ji-Yi Liu

**Affiliations:** 1 Department of Neurology, Affiliated Zhongshan Hospital of Dalian University, Dalian, 116001 Liaoning China; 2 Department of Cardiac Surgery, Affiliated Zhongshan Hospital of Dalian University, Dalian, 116001 Liaoning China; 3 Heart Centre, Affiliated Zhongshan Hospital of Dalian University, Dalian, 116001 Liaoning China

**Keywords:** Aortic stenosis, TAVR, SAVR, Depression, Anxiety, Quality of life, Frailty

## Abstract

**Objective:**

Currently, only a few studies have been conducted on the mental status recovery in elderly aortic stenosis (AS) patients after treatment. How transcatheter aortic valve replacement (TAVR) and surgical aortic valve replacement (SAVR) differentially impinge on the mental status of elderly AS patients is completely unknown. The present prospective study aims to investigate this question by comparing the post-treatment levels of depression and anxiety, quality of life and frailty.

**Methods:**

A total of 120 elderly patients (age above 70) with symptomatic AS were included, where 78 of them were treated with TAVR and 42 of them were treated with SAVR. Levels of depression and anxiety, quality of life and frailty were assessed by the Chinese version of Hospital Anxiety and Depression Scale (HADS), World Health Organization Quality of Life Instrument-Older Adults Module (WHOQOL-OLD) and clinical frailty scale, respectively. Scores were recorded and compared at admission, 1 month, 4 months and 8 months after treatment.

**Results:**

Before treatment, both patient groups had similar baseline characteristics and all mental parameters. During the follow-up period, patients in the TAVR group demonstrated significant improvement in all assessed mental parameters to certain extent compared to the SAVR group. Specifically, frailty was significantly improved in the TAVR-treated patients at all three follow-up time points. Levels of depression and anxiety were significantly improved 8 months after treatment, although the remaining patient number is limited. Quality of life was only significantly improved 1 month after treatment.

**Conclusion:**

TAVR may provide a better mental recovery outcome in elderly AS patients.

## Introduction

Aortic stenosis (AS) is characterized by a narrowing of the aortic valve opening. It is one of the most common and serious valvular heart diseases around the world. The prevalence of AS is approximately 5% in patients above 75 years old, where half of them are at the symptomatic stage that requires immediate treatment [1]. So far, the only causative treatment for symptomatic AS is aortic valve replacement by surgical (SAVR) or transcatheter (TAVR) approach [2]. Before the TAVR method was established, up to one third of the patients have to be devoid from the treatment due to the high surgical risk arose from comorbidities [3]. In the past decade, the TAVR method has developed from a treatment option to the therapy of choice for elderly AS patients regardless of surgical risk [4–8].

Approximately 2% of the global population (about 163 million people) is affected by major depressive disorders (MDD) according to the Global Burden of Diseases, Injuries, and Risk Factors Study performed in 2017 [9]. When it comes to elderly multimorbid patients suffering from cardiovascular diseases, the prevalence rate increases sharply as a result of reduced quality of life and increased risk of unexpected adverse events [10–13]. In German AS patients treated with TAVR, the prevalence of depression is over 30% and is closely associated with an elevated mortality rate [14]. However, another recent study on German population shows that TAVR can effectively reduce depression and anxiety in AS patients and no association between pre-existing depression and anxiety with long-term mortality [15]. Therefore, the impact of TAVR on depression and anxiety in AS patients needs to be verified in other patient populations. Hospital Anxiety and Depression Scale (HADS) questionnaire is one of the most commonly used tools to detect clinical depression. The Chinese version of HADS has been verified as a reliable and valid way to assess the anxiety and depression scale in Chinese cancer patients and their family care givers [16].

In the present study, we aim to compare the impact of SAVR and TAVR treatments on anxiety and depression level, quality of life and clinical frailty in elderly Chinese patient population suffered from symptomatic AS.

## Methods

### Patients

In the present prospective study, 120 symptomatic AS patients treated at Zhongshan Hospital of Dalian University were divided into two groups based on the treatment method (SVAR or TAVR) that they received. Choice of treatment method was decided by clinical surgeons and was agreed by the patients. All patients were above the age of 70. The study was approved by the ethical committee of Zhongshan Hospital of Dalian University and was performed according to the Declaration of Helsinki. Written consent was obtained from all patients. Baseline parameters at the time of hospital admission were recorded for all included patients. Patients were followed up at 1 month, 4 months and 8 months after treatment.

### Assessment of anxiety and depression level, quality of life and clinical frailty

Anxiety and depression levels were detected using the Chinese version of HADS (C-HADS) [16]. The C-HADS contains 14 items that compose two 7-item subclasses, one measuring anxiety and the other measuring depression; which can clearly distinguish anxiety and depression from somatic symptoms. A 4-point Likert-type scoring scale ranging from 0 (no problem) to 3 (big problem) was used for each item. The score for each subclass (anxiety and depression) was obtained by adding up the scores of each individual item within each subclass, resulting in a value between 0 and 21. A score of 7 or less indicates a no-problem case, 8–10 indicates a borderline case and 11 or above indicates a confirmed case.

Quality of life was assessed using the Chinese version of the World Health Organization Quality of Life Instrument-Older Adults Module (WHOQOL-OLD) [17]. The WHOQOL-OLD consists of 24 items that are divided into the following 6 domains: Sensory Abilities (SAB), Autonomy (AUT), Past, Present and Future Activities (PPF), Social Participation (SOP), Death and Dying (DAD) and Intimacy (INT). A 5-point Likert-type scoring scale ranging from 1 to 5 was used for each item, where higher score indicates better quality of life.

Frailty was assessed using the Chinese version of clinical frailty scale (CFS) to summarize the overall level of fitness and predict death or the need for institutional care in elderly people [18]. The CFS evaluates the domains of comorbidity, function, and cognition, to generate a frailty score ranging from 1 to 9 [19]. Level 1–3, defined as very fit, fit and managing well, respectively, indicate degree of fitness prior to the level of risk associated with frailty. Level 4–7, defined as living with very mild, mild, moderate and severe frailty, respectively, indicate clinically meaningful risk of frailty. Level 8 and 9, defined as living with very severe frailty and terminally ill, respectively, reflect people towards the end of life.

### Statistics

Statistical analysis was performed using the SPSS software 27.0 (IBM, Armonk, NY). Statistical significance was determined using the student t test and the one-way ANOVA for continuous data and the Mann–Whitney-U test for categorical data. P < 0.05 was considered as statistical significance.

## Results

### Baseline characteristics

Among the 120 included patients, 42 of them received SAVR and 78 of them received TAVR (20 self-expandable cases and 58 balloon-expandable cases). Embolic protection was not used for any of the TAVR cases. At the time of hospital admission, all baseline characteristics were similar between the two patient groups, including age, sex distribution, medical conditions, New York Heart Association class distribution, C-HADS for depression and anxiety, WHOQOL-OLD for quality of life and CFS for frailty (Table 1). These data suggest that the two patient groups have relatively comparable status before receiving any treatment. It is worth noting that the Society of Thoracic Surgeons (STS) predicted risk of mortality score was marginally higher in the TAVR group (P = 0.055), although both of them fall into the intermediate risk category (score between 4 and 8).

**Table 1:** Baseline characteristics of the patients at hospital admission

Baseline parameter at admission	SAVR (n = 42)	TAVR (n = 78)	P value
Age (year, mean ± SD)	77.6 ± 4.6	78.6 ± 4.8	0.316
Sex (male:female)	28:14	58:20	0.372
Medical condition (no., % of total)			
Diabetes	16 (38.1%)	27 (34.6%)	0.705
Hypertension	37 (88.1%)	71 (91.0%)	0.61
Previous stroke	3 (7.1%)	5 (6.4%)	0.878
Peripheral vascular disease	12 (28.6%)	22 (28.2%)	0.966
Pacemaker	2 (4.8%)	3 (3.8%)	0.811
STS predicted risk of mortality score (mean ± SD)	5.3 ± 2.4	6.2 ± 2.4	0.055
New York Heart Association class (no., % of total)			
II	17 (40.5%)	29 (37.2%)	0.833
III	22 (52.4%)	41 (52.6%)	
IV	3 (7.1%)	8 (10.3%)	
C-HADS depression (no., % of total)			
mean ± SD	7.9 ± 2.9	7.1 ± 3.1	0.156
≤ 7	18 (42.9%)	42 (53.8%)	0.833
8–10	12 (28.6%)	19 (24.4%)	
≥ 11	12 (28.6%)	17 (21.8%)	
C-HADS anxiety (no., % of total)			
mean ± SD	7.5 ± 2.5	6.9 ± 2.6	0.252
≤ 7	21 (50%)	46 (59.0%)	0.194
8–10	14 (33.3%)	27 (34.6%)	
≥ 11	7 (16.7%)	5 (6.4%)	
WHOQOL-OLD (mean ± SD)	60.9 ± 11.5	59.5 ± 12.7	0.552
CFS (no., % of total)			
mean ± SD	4.4 ± 1.8	4.2 ± 1.7	0.471
1–3	15 (35.7%)	32 (41.0%)	0.773
4–7	27 (64.3%)	46 (59.0%)	
8–9	0	0	

### Depression and anxiety status during follow-up

Follow-up questionnaires were performed on all the patients at 1 month, 4 months and 8 months after treatment. In the SAVR group, 2, 2 and 10 patients passed away at the 3 follow-up time points, respectively. In the TAVR group, 3, 5 and 11 patients passed away at the 3 follow-up time points, respectively. Among the 33 deaths, 8 of them were caused by cardiovascular reasons including 3 cases of disabling stroke, 17 of them were caused by non-cardiovascular reasons and 8 of them were caused by unknown reasons. All 3 stroke cases occurred between the 4- and 8-month time points and the patients eventually passed away before being assessed at the 8-month time point.

**Fig. 1:**
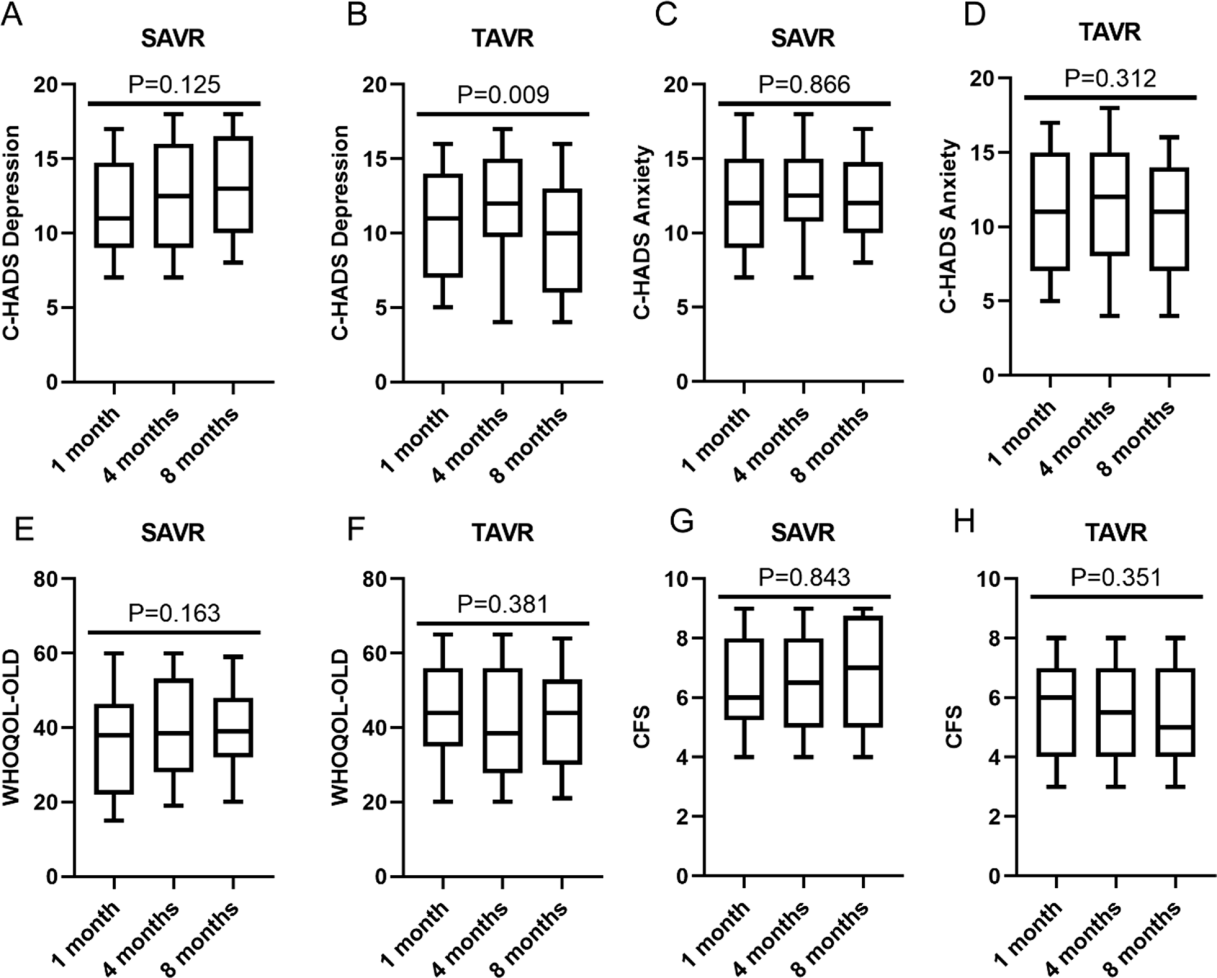
Changes of the mental parameters during the follow-up period

1 month after treatment, WHOQOL-OLD and CFS were significantly improved in the TAVR treated patients (Table 2). For C-HADS anxiety, although the mean values were marginally below the statistically significant threshold (P = 0.069), the number of patients distributed among the 3 scoring categories were significantly different (P = 0.018) (Table 2). Mean values of C-HADS depression were comparable between the 2 patient groups (P = 0.285), whereas the difference in categorical distribution was slightly different (P = 0.082) (Table 2). 4 months after treatment, CFS was significantly improved in the TVAR group (Table 2). C-HADS depression and WHOQOL-OLD were both similar between the 2 groups (Table 2). Same as the previous time point, C-HADS anxiety was only found to be significantly different at the level of categorical distribution (P = 0.04) (Table 2). 8 months after treatment, C-HADS depression, C-HADS anxiety and CFS were all significantly better in the TAVR group, while the WHOQOL-OLD remained comparable (Table 2). When comparing changes in the 4 questionnaires within each treatment group, only C-HADS depression in the TAVR group was significantly improved over time (Fig. 1).

**Table 2:** Mental parameters assessed at 1, 4 and 8 month(s) after treatment

1 month after treatment	SAVR (n = 40)	TAVR (n = 75)	P value
C-HADS depression (no., % of total)			
mean ± SD	11.4 ± 3.1	10.7 ± 3.6	0.285
≤ 7	4 (10%)	18 (24%)	0.082
8–10	14 (35%)	15 (20%)	
≥ 11	22 (55%)	42 (56%)	
C-HADS anxiety (no., % of total)			
mean ± SD	12.2 ± 3.4	10.9 ± 3.8	0.069
≤ 7	2 (5%)	20 (26.7%)	0.018
8–10	12 (30%)	16 (21.3%)	
≥ 11	26 (65%)	39 (52%)	
WHOQOL-OLD (mean ± SD)	34.7 ± 14.0	44.1 ± 12.4	< 0.001
CFS (no., % of total)			
mean ± SD	6.6 ± 1.6	5.5 ± 1.8	0.002
1–3	0	15 (20%)	0.017
4–7	29 (72.5%)	49 (65.3%)	
8–9	11 (27.5%)	11 (14.7%)	
4 months after treatment	SAVR (n = 38)	TAVR (n = 70)	P value
C-HADS depression (no., % of total)			
mean ± SD	12.5 ± 3.7	11.9 ± 3.9	0.445
≤ 7	4 (10.5%)	14 (20%)	0.216
8–10	9 (23.7%)	9 (12.9%)	
≥ 11	25 (65.8%)	47 (67.1%)	
C-HADS anxiety (no., % of total)			
mean ± SD	12.5 ± 3.1	11.4 ± 4.2	0.147
≤ 7	2 (5.3%)	16 (22.9%)	0.04
8–10	12 (31.6%)	13 (18.6%)	
≥ 11	24 (63.2%)	41 (58.6%)	
WHOQOL-OLD (mean ± SD)	39.6 ± 13.1	41.1 ± 14.0	0.586
CFS (no., % of total)			
mean ± SD	6.5 ± 1.7	5.6 ± 1.7	0.006
1–3	0	11 (15.7%)	< 0.001
4–7	26 (68.4%)	7 (10%)	
8–9	12 (31.6%)	52 (74.3%)	
8 months after treatment	SAVR (n = 28)	TAVR (n = 59)	P value
C-HADS depression (no., % of total)			
mean ± SD	13.1 ± 3.4	9.9 ± 3.8	< 0.001
≤ 7	0	20 (33.9%)	0.007
8–10	8 (28.6%)	12 (20.3%)	
≥ 11	20 (71.4%)	27 (45.8%)	
C-HADS anxiety (no., % of total)			
mean ± SD	12.1 ± 2.8	10.3 ± 3.7	0.024
≤ 7	0	16 (27.1%)	0.017
8–10	9 (32.1%)	9 (15.3%)	
≥ 11	19 (67.9%)	44 (74.6%)	
WHOQOL-OLD (mean ± SD)	40.0 ± 11.9	42.3 ± 12.9	0.423
CFS (no., % of total)			
mean ± SD	6.8 ± 1.6	5.2 ± 1.8	< 0.001
1–3	0	14 (23.7%)	< 0.001
4–7	9 (32.1%)	37 (62.7%)	
8–9	19 (67.9%)	8 (13.6%)

Taken together, these results show that TAVR has better outcome on improving the depression and anxiety status of AS patients, compared to SAVR.

## Discussion

Mental disorders, especially depression and anxiety, have high impact on elderly patients suffering from cardiovascular diseases as co-morbidities. Majority of the published studies focus on the mental status of TAVR-treated patients alone, but not comparing between the SAVR and TAVR treatment. A previous prospective study performed on German population shows that health-related quality of life could be significantly improved in elderly patients with aortic valve diseases after TAVR treatment [14]. Another recent German study shows that TAVR could reduce depression and anxiety in AS patients who demonstrated pathologic baseline values in HADS depression and anxiety [15]. A multicenter prospective study was performed on Canadian population to check the prevalence of depression and its association with all-cause mortality in elderly patients who underwent either SAVR or TAVR [20]. It shows that 31.5% of the patients experienced depression at baseline accompanied by a higher risk of short-and midterm mortality. However, this study employed the 5-item version of the Geriatric Depression Scale Short Form for depression assessment, which is methodologically different form the HADS used in the present study. The death rate of our patient cohort was relatively higher than previous studies. However, only less than 25% of the deaths (8/33) were caused by identified cardiovascular reasons. Therefore, differences in patients’ basic health state might be the reason that leads to the discrepancies in death rate.

To the best of our knowledge, our study is the first one to compare the mental status between TAVR and SAVR treated elderly Chinese patients with symptomatic AS. In general, we show that TAVR has a better treatment outcome on patients’ mental status from 1 month after treatment compared to SAVR. At baseline, 24.2% (29 out of 120), 10% (12 out of 120) and 60.8% (73 out of 120) of the patients were classified as the pathological categories of depression (C-HADS depression ≥ 11), anxiety (C-HADS anxiety ≥ 11) and frailty (CFS ≥ 4), respectively. Compared to the German population, our baseline percentage of patients with anxiety is much lower (28.6%), whereas the baseline percentage of patients with depression is similar (23.6%) (15). By definition, anxiety is an active feeling of unease, including worry and fear; while depression negatively affects a person’s feeling. Considering the generally laid-back personality of Chinese people, the discrepancy might be explained by the personality difference of the two patient populations.

Compared to SAVR, TAVR is a less invasive procedure that may reduce patients’ anxiety and depression before and after treatment. However, anxiety and depression associated with aortic-valve replacement in elderly patients are rather complicated. Patients’ neurological status will not only be affected by their wound healing progress, but also other factors including changes in life routine, disappoint and discouragement of the life ahead, reduced key neurotransmitters in the brain, family history of depression, previous traumatic life events, other accompanied medical conditions etc. Therefore, the findings of our study are not a simple reflection of the wound healing condition of the patients, but a reflection of all the potential factors mentioned above, which support TAVR as a better option for aortic-valve replacement from a neurological point of view.

In general, limited study has been reported on the treatment-associated dynamics in depression, anxiety, quality of life and frailty. On the other hand, other intervention methods have been shown to alleviate mental-related symptoms. For example, depression could be suppressed by exercise training in addition to therapeutic treatment in heart failure patients [21, 22]. Moreover, a decrease in depression following neither TAVR nor SAVR treatment was observed in AS patients [20]. Therefore, psychological interventions might play a role in relieving the depression symptoms and thereby improve patients’ recovery outcome. This hypothesis is supported by several studies in patients with coronary artery diseases [23, 24].

One limitation of the present study is its single-institution nature and limited number of patients, especially for the 8 month time point, which make the study underpowered to draw definite conclusions. Future multicenter studies with more variety and number of patients are needed to confirm our observation. Although the Chinese version of tools to assess depression, anxiety, quality of life and frailty used in our study have been well validated in Chinese patient population, there are other tools that could be used for these purposes instead. Therefore, future study employing other assessment tools are also needed to solidify our findings.

## Conclusions

In summary, this is the first study to compare the mental status between TAVR and SAVR treated elderly Chinese AS patients. We show that TAVR has a trend of better outcome on depression, anxiety, quality of life and frailty of these patients from as soon as 1 month after treatment. These data suggest that TAVR seems to be a better treatment option for elderly patients in the mental status recovery perspective.

## Data Availability

Not applicable.
